# Identifying stroke diagnosis-related features from medical imaging reports to improve clinical decision-making support

**DOI:** 10.1186/s12911-022-02012-3

**Published:** 2022-10-20

**Authors:** Xiaowei Xu, Lu Qin, Lingling Ding, Chunjuan Wang, Meng Wang, Zixiao Li, Jiao Li

**Affiliations:** 1 Institute of Medical Information, Chinese Academy of Medical Sciences, Peking Union Medical College, Beijing, China; 2 Beijing Tiantan Hospital, Capital Medical University, Beijing, China

**Keywords:** Ischaemic stroke, High-Signal intensity regions, DWI, MRI reports, Fine-grained entity typing

## Abstract

**Background:**

Medical imaging reports play an important role in communication of diagnostic information between radiologists and clinicians. Head magnetic resonance imaging (MRI) reports can provide evidence that is widely used in the diagnosis and treatment of ischaemic stroke. The high-signal regions of diffusion-weighted imaging (DWI) images in MRI reports are key evidence. Correctly identifying high-signal regions of DWI images is helpful for the treatment of ischaemic stroke patients. Since most of the multiple signals recorded in head MRI reports appear in the same part, it is challenging to identify high-signal regions of DWI images from MRI reports.

**Methods:**

We developed a deep learning model to automatically identify high-signal regions of DWI images from head MRI reports. We proposed a fine-grained entity typing model based on machine reading comprehension that transformed the traditional two-step fine-grained entity typing task into a question-answering task.

**Results:**

To prove the validity of the model proposed, we compared it with the fine-grained entity typing model, of which the F1 measure was 5.9% and 3.2% higher than the F1 measures of the models based on LSTM and BERT, respectively.

**Conclusion:**

In this study, we explore the automatic identification of high-signal regions of DWI images from the description part of a head MRI report. We transformed the identification of high-signal regions of DWI images to an FET task and proposed an MRC-FET model. Compared with the traditional two-step FET method, the model we proposed not only simplifies the task but also has better performance. The comparable result shows that the work in this study can contribute to improving the clinical decision support system.

## Introduction

The application of artificial intelligence technologies to medical images is the foundation of intelligent clinical decision-making support. Currently, a medical imaging report consists of not only timely patient data but also evidence-based approaches and multiparametric computational and analytical methods, including patient-specific and population-based clinical, laboratory, genomic, demographic and quantitative data.

**Fig. 1:**
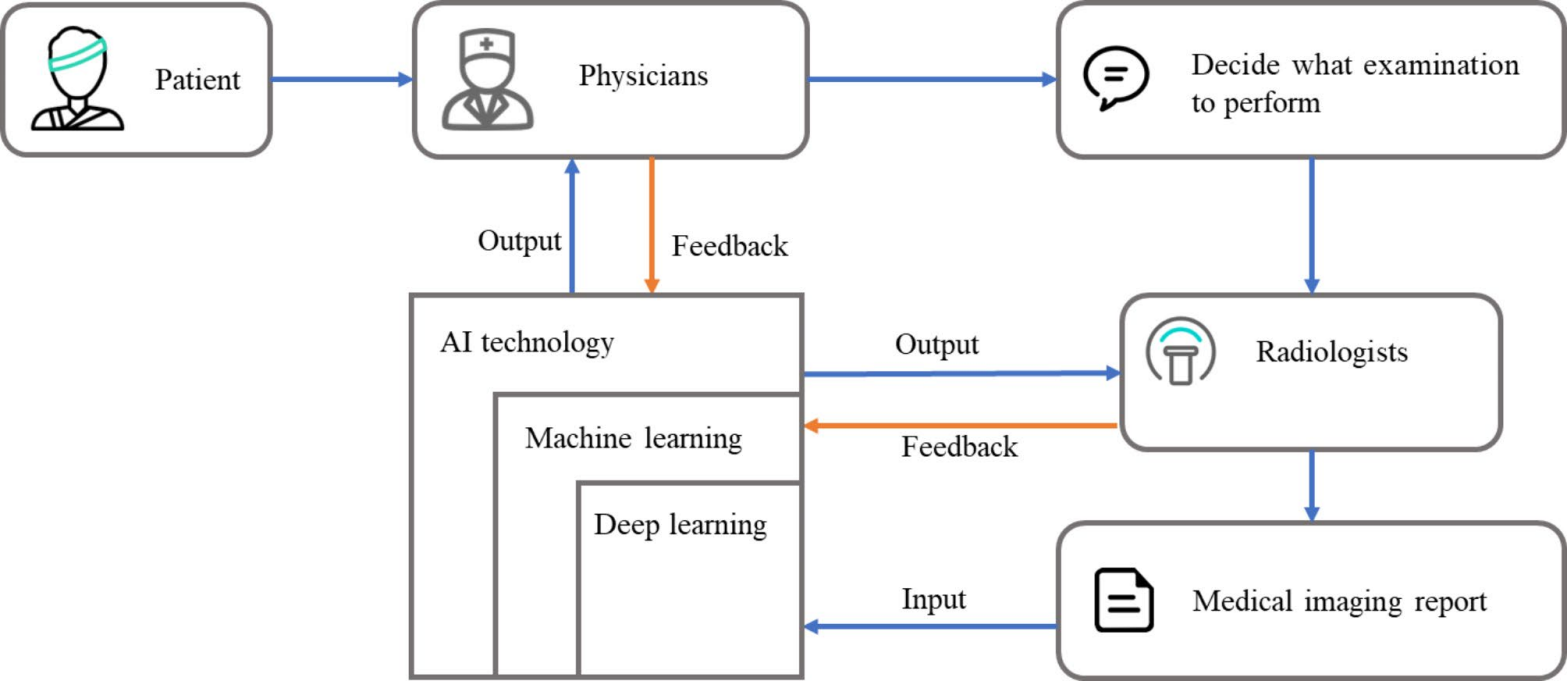
Information flow of a medical imaging report

In clinical practice, when a patient arrives at the hospital, the physician will decide what examination to perform and request for imaging study based on the patient’s information. The order will be transmitted to the radiologist through the hospital information system. Radiologists usually manually identify the important information needed case by case and subsequently generate the image report. The manual film-reading method is labour intensive and inefficient. Studies report that, in some cases, an average radiologist must interpret one image every 3–4 s in an 8-hour workday to meet workload demands [1]. As radiology involves visual perception as well as decision-making under uncertain conditions [2], errors are inevitable–especially under such constrained conditions. At the same time, the lack of standards in the record, such as the phraseology difference and writing habits of radiologists, leads to the misunderstanding between the radiologist and the physician. With the development and application of deep learning technology in medical imaging, different artificial intelligence technologies have been applied to computer-aided medical film reading [3][4], management options with probabilities grounded in evidence-based appropriate-use criteria can be presented to physicians so they can make better clinical decisions. Meanwhile, the feedback of the physicians and the radiologists will help to improve the performance of the deep learning algorithms, as shown in Fig. 1.

MRI of the head, as a key category of medical imaging reports, using a powerful magnetic field, radio waves and a computer to produce detailed pictures of the brain and other cranial structures, is widely used in clinics [5]. Common head MRI sequences T1-weighted imaging (T1WI), T2-weighted imaging (T2WI), fluid attenuated inversion recovery (FLAIR), DWI and so on, among which DWI is used to detect the Brownian motion of water molecules in human tissues [6]. When the diffusion of water molecules in tissues is normal, the image has an equal signal, while when diffusion is limited, abnormally high-intensity signals are observed in DWI images. The signal intensity of DWI images depends on T2WI and the apparent diffusion coefficient (ADC), and the formula is as follows:

$${I}_{DWI}\propto {I}_{T2WI}\times {e}^{-b\times ADC}$$

In the above formula, b represents the diffusion sensitivity factor. The larger b is, the greater the detection ability of the diffusion function. The greater the contrast between the lesion and normal tissue is, the higher the sensitivity. The causes of high signal intensity in DWI images are the prolongation of the T2WI signal (such as T2 shine-through) and the decrease in ADC. Many diseases, such as ischaemic stroke, brain tumours, brain abscesses, and lymphoma, show high signals in DWI. High signal intensity in DWI is the most sensitive sequence for detecting the infarct focus of acute ischaemic stroke, also known as the “stroke sequence“[7][8][9]. Therefore, the identification of high-signal regions in DWI is helpful for the treatment of ischaemic stroke patients.

Woo et al.[10] used convolutional neural networks (CNNs) to identify high-signal regions in DWI images to automatically segment acute ischaemic lesions and compared them with conventional algorithms. The results showed that the CNN algorithm had superior performance compared to the other conventional algorithms. Based on previous studies, Chang et al.[11] used the advanced 3D U-Net neural network to automatically identify high-signal regions from DWI images, and the intraclass correlation coefficient of the recognition results on the test set reached 0.974. Meanwhile, natural language processing (NLP) technologies have been applied to identify subjects from radiology reports. Kim et al.[12] used NLP and machine learning algorithms to identify acute ischaemic stroke from brain MRI reports. Carrodeguas E et al.[13] used traditional machine learning and deep learning models to identify follow-up recommendations in radiology reports. Kang et al.[14] used NLP technology to identify incidental lung nodules in radiology reports to assess management recommendations. However, as far as we know, there is no research on the automatic extraction of the high-signal regions of DWI images from head MRI reports. The application of natural language processing technology in the medical field, especially in the structured processing of MRI reports, shows great advantages[15] and provides an opportunity to obtain the high-signal regions of DWI images from MRI reports.

**Fig. 2:**
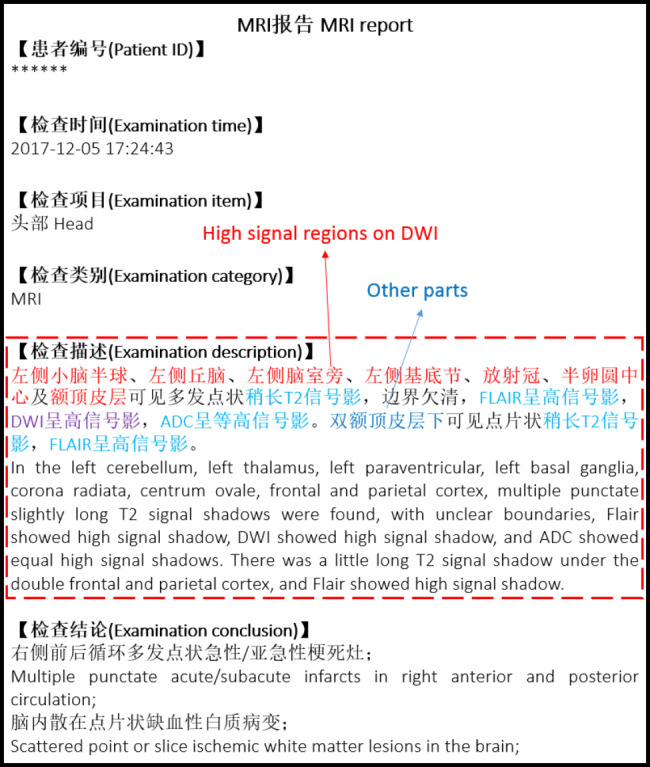
Example head MRI report. The paragraph in the dotted box is the detailed examination description. The text in red means the high signal regions on DWI, and the text in dark blue means the other region parts. The text in purple means the statement of DWI, while the text in light blue means statement of the other modalities

The head MRI reports used in this study are from the electronic medical record (EMR) system of Beijing Tiantan Hospital, Capital Medical University. An example of an MRI report is shown in Fig. 2. In traditional medical natural language processing tasks, named-entity recognition technology is usually used to identify diseases, body parts, etc.[18, 16]). However, as the report in Fig. 2 shows, an MRI report records not only the high-signal regions of DWI images but also the high-signal regions from FLAIR, long-signal regions from T2WI, and some other regions but also high/long-signal regions from other MRI sequences, which causes considerable linguistic interference when using NER technologies to identify the high-signal regions of DWI images. Owing to the above factors, we transform the task of identifying the high-signal regions of DWI images to a fine-grained entity typing task.

## Related work

**Fig. 3:**
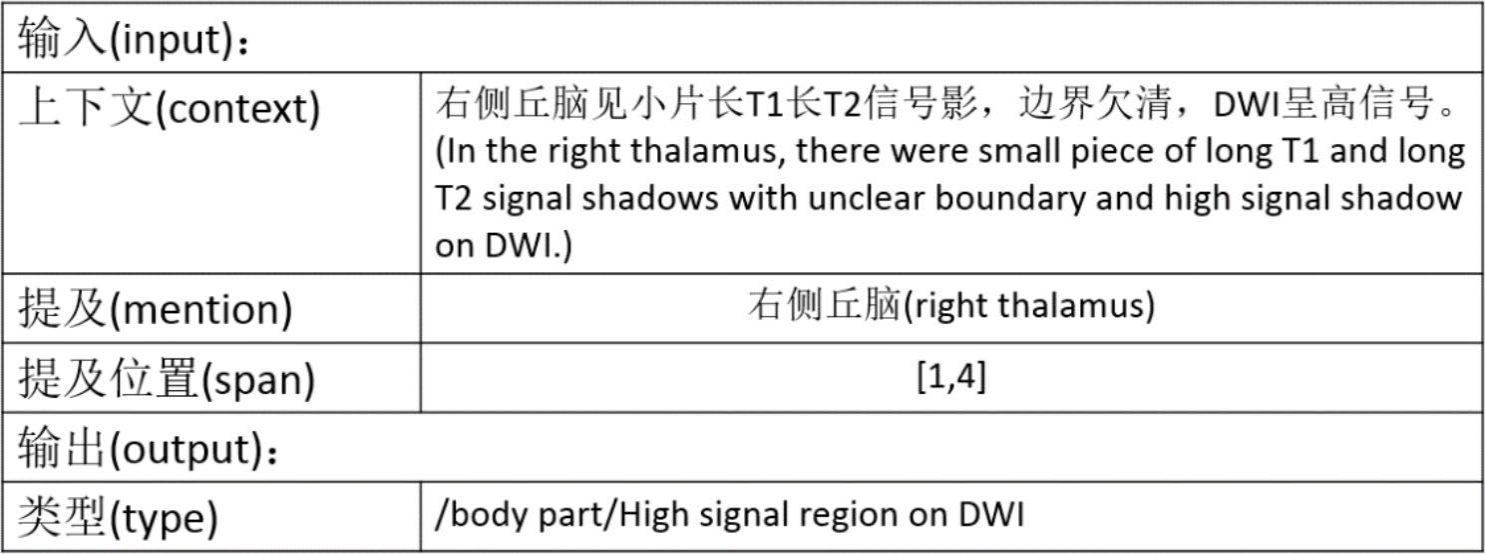
Example of fine-grained entity typing task input and output

According to the different granularities of entity types, there are mainly two kinds of tasks in academia: named-entity recognition (NER) [17] [18]and fine-grained entity typing (FET) [19]. The former task extracts the mention from the text and predicts its type in the context, which is usually coarse-grained, such as diseases and body parts. Therefore, the subtask of predicting the type of mentions can be regarded as coarse-grained entity typing. The latter task predicts the entity type according to the mention type given. For example, the high-signal region in DWI is a subtype of the body part. Figure 3 shows the input and output of a traditional FET system. We therefore define the task as a combination of NER and FET tasks.

From the output of the example, we can divide the task of high-signal region recognition in DWI into two stages: first, recognizing all the body parts in an MRI report, namely, an NER task, and second, dividing the identified parts into high-signal regions of DWI images and other regions, namely, an FET task.

**Fig. 4:**
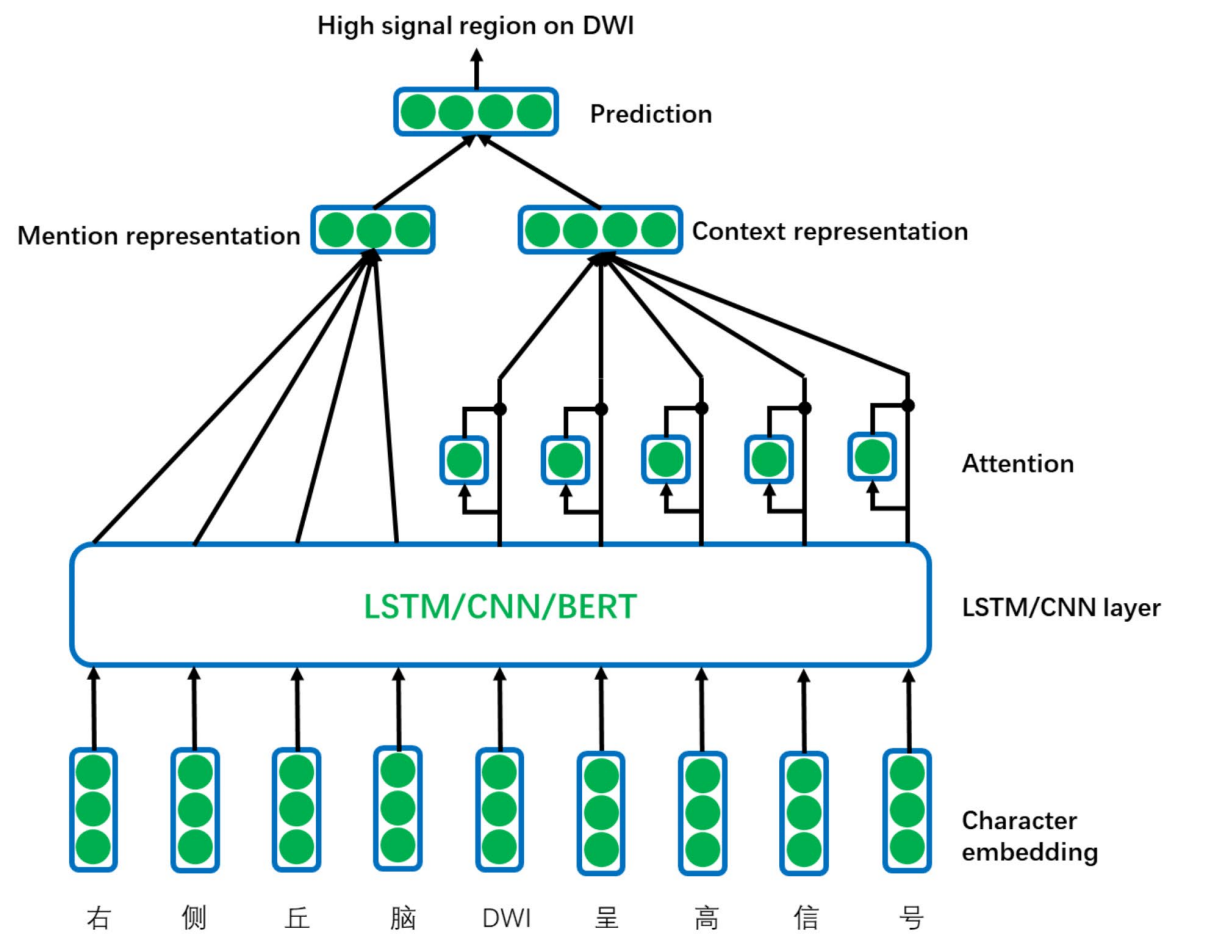
General structure of the fine-grained entity typing model

The BiLSTM-CRF model is one of the most widely used technologies in the named-entity recognition task [19–21]. In comparison with the CRF, BiLSTM and BiLSTM-CRF models in English EMRs, the BiLSTM-CRF model performed best[22]. With the development of pretraining language models such as BERT[23], the performance of NLP tasks has been further improved. Zhang et al.[24] used the BERT-based BiLSTM-CRF model to identify breast cancer from progress notes and discharge summaries of 100 breast cancer patients, for which the F1 score reached 93.53%, better than that of the BiLSTM-CRF model. On the other hand, FET is mainly used in the general field and rarely in the medical field. The essence of fine-grained name-entity typing is a multilabel classification problem. When the entity and its context are known, the entity can be classified into a specific category. The commonly used structure of the fine-grained named entity typing model is shown in Fig. 4. Generally, it is a three-layer structure. The first layer is the text input layer, which transforms the text into the form of a character vector or word vector as the input of the second layer. The second layer is the representation layer of entity and context, and the CNN, LSTM, and BERT models and attention mechanism are commonly used for feature extraction to obtain vector representations of an entity and entity context, respectively. The third layer is the prediction layer, which predicts the entity category[25][26]. Lee et al. [27] compared the performances of fine-grained entity typing models based on LSTM and BERT on a Chinese corpus, and the results showed that the performance of the latter is much better than that of the former. If we simply combine the NER model and the FET model, errors will accumulate, which will lead to poor performance. Li et al.[28] recently proposed a machine reading comprehension method to transform the traditional NER task (sequence labelling) into a question-answering task. Since the problem itself could provide additional information, this method could be introduced into an FET task. Therefore, we used the machine reading comprehension method to identify the high-signal regions of DWI images in this study.

## Method

### Dataset

The head MRI reports used in this study are from Beijing Tiantan Hospital, Capital Medical University. There were 2527 head MRI reports of 2527 patients, as shown in Fig. 2. MRI reports included patient ID, examination time, examination item, examination category, examination description and examination conclusion. The description of the DWI signal is mainly recorded in the examination description. Therefore, we chose the description part of the report to identify the high-signal regions of DWI images. After removing spaces and line breaks and converting upper-case English letters to lower-case English letters, we used the open-source software BRAT[29] to annotate the data. The annotation process is divided into two stages. The first stage is to annotate all body parts. The semiautomatic annotation method was adopted. First, 200 reports were manually annotated. All body parts appearing in the description part of MRI reports were annotated in the 200 reports (without identifying the high-signal regions of DWI images from other parts). The double-blind annotation method has been adopted for the annotation of body parts in the 200 MRI reports. Two junior radiologists are involved in the first round of annotation. For the inconsistent annotation results, an audit expert senior radiologist, with ten-years working experience, was invited to compare and determine the final answer, which acts as golden standard of the annotation results. From the feedback of the audit expert, the main inconsistency between the two annotators is the omission of some body parts.

Then, we trained a BiLSTM-CRF model to identify body parts of MRI reports with the 200 annotated reports, which was performed to automatically annotate the remaining 2327 reports. To ensure the accuracy of the corpus, the same audit expert was invited to check the annotation results identified by the BiLSTM-CRF model. The audit expert modified 331 annotated body parts and added 43 body parts annotations. This semiautomatic annotation method greatly reduces the annotation workload in the first stage. Based on the first stage, we manually annotated parts in the description part of the MRI report as the high-signal regions of DWI images and other parts in the second stage. After annotation, clinical experts were also invited to review the corpus annotation and they approved the annotation results. Therefore, we obtained the final labelled corpus.

A total of 4369 high-signal regions of DWI images and 19260 other parts were annotated in the annotated corpus set. The average numbers of high-signal regions of DWI images and other parts in each examination description part of the report were 2 and 8, respectively. 90% of the examination descriptions in the reports did not contain any parts. The average length of each examination description was 170 Chinese characters, and the longest one consisted of 525 Chinese characters, while the shortest one consisted of only 2 Chinese characters. Then, we divided the corpus, all the annotated examination descriptions of the reports, into a training set and a testing set according to a ratio of 8:2. The training set contained 2022 examination descriptions of reports, including 3506 high-signal regions of DWI images and 15352 other parts. The testing set contained 505 examination descriptions of reports, including 863 high-signal regions of DWI images and 3908 other parts. The tagging scheme we used in this study is BIOES[30], in which B stands for ‘beginning’ (signifying the beginning of an entity), I stands for ‘inside’ (signifying that the character is inside an entity), O stands for ‘outside’ (signifying that the character is just a regular character outside of an entity), E stands for ‘end’ (signifying that the character is the end of an entity), and S stands for ‘singleton’ (signifying that the single character is an entity).

### Model

We proposed an FET model based on the machine reading comprehension (MRC-FET) method, which simplifies the original two-step fine-grained entity typing task into one step and compared the result with the work of Lee et al.[27] The traditional fine-grained entity typing task consists of two steps: NER and entity typing.

**Fig. 5:**
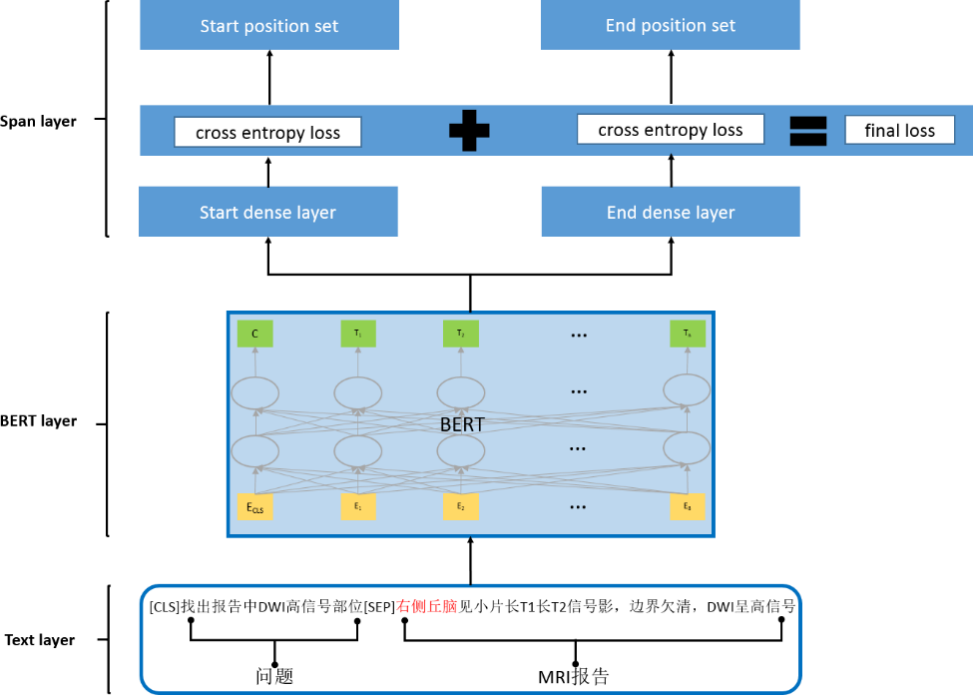
Structure of MRC-FET

The structure of MRC-FET is shown in Fig. 5. The model consists of three layers. The first layer is the text layer. In the MRC-FET experiment, the question is “找出报告中DWI 高信号部位” (to find high-signal regions of DWI images in the MRI report), and the answer is high-signal regions of DWI images. First, we transform the MRI report into the format that the machine reading comprehension method in BERT needs, such as $\left( CLS,{q}_{1},{q}_{2},\dots ,{q}_{m },SEP,{x}_{1},{x}_{2},\dots ,{x}_{n }\right)$, in which ${q}_{i}$ is the question character and ${x}_{i}$ is the MRI report character. Since the question shows what the experiment is to do, it can provide more information than the traditional fine-grained entity typing methods. The second layer is the BERT layer, and the output is matrix $E\in {R}^{n\times d}$, where n is the length of the MRI report description and d is the vector dimension of the last layer of the BERT model. The third layer is the span layer, which is mainly used to predict the start and end positions of the high-signal regions of DWI images in MRI reports according to the output of the BERT model. Start position prediction predicts the possibility that each character in the MRI report is the start position of the high-signal region of DWI images based on the matrix $E$ output by BERT. The calculation formula is as follows:

$${\text{P}}_{\text{s}\text{t}\text{a}\text{r}\text{t}}=\left( {\text{s}\text{o}\text{f}\text{t}\text{m}\text{a}\text{x}}_{each row}(\text{E}\bullet {\text{T}}_{start}\right))\in {R}^{n\times 2}$$

where ${\text{T}}_{start}$ is the parameter that the model needs to learn. Each line of ${\text{P}}_{\text{s}\text{t}\text{a}\text{r}\text{t}}$ represents the possibility that each character in the MRI report is the start position of the high-signal region of the DWI image. For example, if the first line of ${\text{P}}_{\text{s}\text{t}\text{a}\text{r}\text{t}}$ is [0.3,0.7], the first character is not the start position of the high-signal region of the DWI image. If the second line of ${\text{P}}_{\text{s}\text{t}\text{a}\text{r}\text{t}}$ is [0.8,0.2], the second character may be the start position of the high-signal region of the DWI image. Similarly, end position prediction predicts the possibility that each character in the MRI report is the end position of the high-signal region of the DWI image based on the matrix $E$ output by BERT. The calculation formula is as follows:

$${\text{P}}_{\text{e}\text{n}\text{d}}=\left( {\text{s}\text{o}\text{f}\text{t}\text{m}\text{a}\text{x}}_{each row}(\text{E}\bullet {\text{T}}_{end}\right))\in {R}^{n\times 2}$$

where ${\text{T}}_{end}$ is the parameter that the model needs to learn. Each line of ${\text{P}}_{\text{e}\text{n}\text{d}}$ represents the possibility that each character in the MRI report is the end position of the high-signal region of the DWI image. Then, the argmax function acts on ${\text{P}}_{\text{s}\text{t}\text{a}\text{r}\text{t}}$ and ${\text{P}}_{\text{e}\text{n}\text{d}}$, and two 0–1 sequences of length n ${\text{I}}_{\text{s}\text{t}\text{a}\text{r}\text{t}}$ and ${\text{I}}_{\text{e}\text{n}\text{d}}$ are obtained. If the i-th position is 1, then the i-th character may be the start or end position of the high-signal region of the DWI image.

$${\text{I}}_{\text{s}\text{t}\text{a}\text{r}\text{t}}=\left( {\text{a}\text{r}\text{g}\text{m}\text{a}\text{x}}_{each row}\left({\text{P}}_{start}\right)\right)$$

$${\text{I}}_{\text{e}\text{n}\text{d}}=\left( {argmax}_{each row}\left({P}_{end}\right)\right)$$

After obtaining ${\text{I}}_{\text{s}\text{t}\text{a}\text{r}\text{t}}$ and ${\text{I}}_{\text{e}\text{n}\text{d}}$, we match the start position and the end position according to the order of characters, and then, we obtain the position of the high-signal region of the DWI image in the MRI report. For example, if ${\text{I}}_{\text{s}\text{t}\text{a}\text{r}\text{t}}$ is [0,1,0,0,0,0,0,1,0,0] and ${\text{I}}_{\text{e}\text{n}\text{d}}$ is [0,0,0,1,0,0,0,0,0,0,0,0,1], then the locations of the high-signal regions of the DWI images are [2, 4] and [7, 10], respectively, because the start positions of the high-signal regions of the DWI images are 2 and 7 in ${\text{I}}_{\text{s}\text{t}\text{a}\text{r}\text{t}}$ and the end positions are 4 and 10 in ${\text{I}}_{\text{e}\text{n}\text{d}}$, respectively. Thus, the positions of the high-signal regions of the DWI images in the description part of the MRI report are obtained by matching the start and end positions in sequence.

In the model training stage, we used two losses, the start position loss and the end position loss, which are defined as follows:

$${\text{L}}_{\text{s}\text{t}\text{a}\text{r}\text{t}}=\text{C}\text{r}\text{o}\text{s}\text{s}\_\text{E}\text{n}\text{t}\text{r}\text{o}\text{p}\text{y}({\text{P}}_{\text{s}\text{t}\text{a}\text{r}\text{t}},{\text{Y}}_{\text{s}\text{t}\text{a}\text{r}\text{t}})$$

$${\text{L}}_{\text{e}\text{n}\text{d}}=\text{C}\text{r}\text{o}\text{s}\text{s}\_\text{E}\text{n}\text{t}\text{r}\text{o}\text{p}\text{y}({\text{P}}_{\text{e}\text{n}\text{d}},{\text{Y}}_{\text{e}\text{n}\text{d}})$$

The cross entropy between the predicted and real results is determined, and then, the start position loss is added to the end position loss to obtain the final loss.

All methods were performed in accordance with the relevant guidelines and regulations.

## Experiment

### Evaluation criteria

In this study, we used the precision, recall and F1 measure to evaluate the models. We took the result predicted by the model on the test set as Pre=[${p}_{1}, {p}_{2},\dots ,{p}_{i}$] and the result of manual annotation as Gol=[${g}_{1}, {g}_{2},\dots ,{g}_{j}$]. The set element is a mention of the high-signal regions of DWI images and is represented by triple $<{d,pos}_{start}, {pos}_{end}>$, where d is the serial number of the corpus, and ${pos}_{start}$ and ${pos}_{end}$ represent the start and end positions of high-signal regions in DWI images, respectively. When

$${p}_{i}.d={g}_{j}.d$$

$${p}_{i}.{pos}_{start}={g}_{j}.{pos}_{start}$$

$${p}_{i}.{pos}_{end}={g}_{j}.{pos}_{end}$$

the equation ${p}_{i}={g}_{j}$ holds, and the precision, recall and F1 measure are calculated as follows:

$$\text{P}=\frac{\left|intersection\right(\text{P}\text{r}\text{e},\text{G}\text{o}\text{l}\left)\right|}{\left|\text{P}\text{r}\text{e}\right|}$$

$$\text{R}=\frac{\left|intersection\right(\text{P}\text{r}\text{e},\text{G}\text{o}\text{l}\left)\right|}{\left|\text{G}\text{o}\text{l}\right|}$$

$$\text{F}1=\frac{2\text{P}\text{R}}{\text{P}+\text{R}}$$

### Experimental parameters

The experimental code of this study is based on the PyTorch deep learning framework. The parameter settings of MRC-FET, BERT-BiLSTM-CRF, FET based on LSTM (LSTM-FET), and the FET model based on BERT (BERT-FET) are shown in Table 1, and the parameters except the character length in the last two models are consistent with those in Lee et al.[27]. Through our statistics, the character length of over 95% of MRI reports we obtained was less than 400. Then, we set the character length to 400.

**Table 1:** Model parameter settings

Model	Character length	learning rate	batch size	epoch	optimizer
MRC-FET	400	0.00003	32	5	Adam
BERT-BiLSTM-CRF	400	0.00003	32	5	Adam
BiLSTM-FET	400	0.001	256	15	Adam
BERT-FET	400	0.00003	32	5	Adam

## Results

In the research of Jagannatha et al.[22] and Zhang et al.[24], the BERT and BiLSTM models were widely used in NER tasks and had good performance. Therefore, in the comparative experiments, the BERT-BiLSTM-CRF model identifying all parts in the MRI report description part was used in the first step. The BiLSTM-based FET model and BERT-based FET model were used in the entity typing task in the second step.

**Fig. 6:**
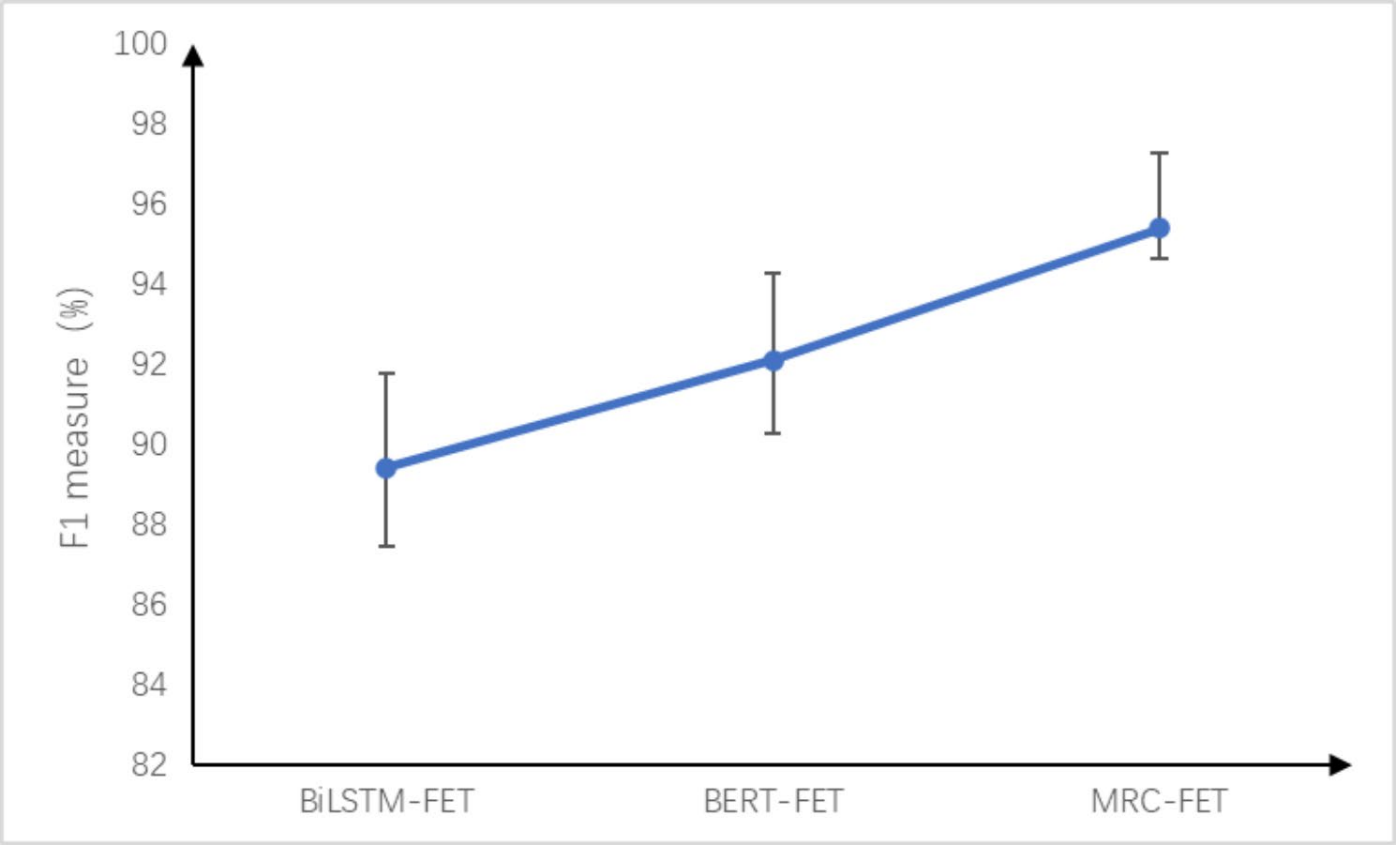
Error bars of the three models

Table 2 shows that the MRC-FET model proposed in this study has the best performance on the testing set, with precision, recall and F1 measure values reaching 97.27%, 93.62% and 95.41%, respectively. The F1 measure is 5.9% and 3.2% higher than that of BiLSTM-FET and that of BERT-FET, respectively. We also report the error bars of the three models in Fig. 6. It can be seen that the error bar of the MRC-FET model we proposed is the shortest, which indicates that the model has better identification performance and stability.

The result shows that the MRC-FET model can not only simplify the task of identifying high-signal regions in DWI but also improve the performance.

**Table 2:** Results of identifying high-signal regions in DWI

Model	Precision (%)	Recall (%)	F1 measure (%)
BiLSTM-FET	86.39	92.7	89.44
BERT-FET	93.44	90.84	92.12
MRC-FET	97.27	93.62	95.41

## Discussion

We analyse the reasons why the MRC-FET model performs well. First, the MRC-FET model is based on the BERT pretrained model, which can obtain bidirectional representations of deep-seated text and greatly improve the performance of downstream natural language processing tasks. Second, it draws lessons from the question-answering method in machine reading comprehension tasks and can clearly describe the task in question, transforming the traditional NER task (sequence labelling) into a question-answering task, “找出报告中DWI 高信号部位” (to find high-signal regions of DWI images in the MRI report), with the high-signal regions of DWI images as answers, which contributes to deal with the considerable linguistic interferences when using traditional NER technologies to identify the high-signal regions of DWI images, and also provides a new idea to solve fine-grained entity typing tasks. Finally, the MRC-FET model simplifies the traditional FET task and avoids the error accumulation of the original models.

In the comparative experiment, the two stages of BERT-FET are both based on the BERT pretrained model. Before the experiment, we speculated that the F1 scores of the BERT-FET and MRC-FET models on the testing set should have a small difference, which was 3.2%. We then conducted an experiment to analyse the reasons for the large difference. We used the MRC-FET model to identify all parts in the description part of the MRI report in the first step of fine-grained entity typing. In MRC-FET, we changed the question to “to find all parts in the MRI report” (origin is “to find high-signal regions of DWI images in the MRI report”). The precision, recall and F1 measure of the MRC-FET model were 95.93%, 96.70% and 96.31%, respectively, while those of the BERT-BiLSTM-CRF model were 94.91%, 93.37%, 94.13%, respectively. The F1 measure of the BERT-BiLSTM-CRF model is 2.18% worse than that of the MRC-FET model in recognizing all parts in the first step, which is the main reason why the performance of body part recognition is poor in the first step. On the other hand, the MRC-FET model proposed in this study can not only achieve good performance in the FET task but also perform well in the traditional NER task, which unifies NER and FET tasks. In addition, introducing machine reading comprehension methods into sequence annotation can inspire the development of other natural language processing tasks.

Finally, we analysed the reasons for the error in MRC-FET prediction, which can be divided into three categories, as shown in Table 3. The first is due to the broken writing of MRI reports; in “脑桥双底节放射冠双额”, the standard writing is “脑桥、双底节、放射冠、双额”, the model only identifies the “脑桥”, the “双底节”, “放射冠” and “双额” were not identified, “双侧丘脑底节放射冠” is a similar situation, the standard writing is “双侧丘脑、底节、放射冠”, and the model also only identified the “双侧丘脑”. To address this issue, we can expand the dataset or strictly regulate the writing of MRI reports from the source to make improvements. The second reason is semantic similarity. For example, one of the testing sets describes “DWI等高信号” (“DWI等信号”) as its similarity with the high-signal region of the DWI image, and the model identifies some parts from the report that do not have to be identified. For this error, we expect a much stronger language model than BERT to appear in the future to help understand the context. The third type is caused by the few appearances of words in the corpus, such as “右侧脑室体部旁脑白质”, which only appears once in the training set and results in incomplete learning of the training model and poor scores in the testing set. For these errors, we can expand the scale of the training set and add more regular expressions in future research.

**Table 3:** Error examples of the MRC-FET model’s prediction results on the testing set

Error type	Example		
	Origin text	Gold standard	Prediction
broken writing	脑桥双底节放射冠双额	脑桥,双底节,放射冠,双额	脑桥
semantic similarity	xxxDWI等高信号xxx	None	Several regions
rare	右侧脑室体部旁脑白质	右侧脑室体部旁脑白质	None

## Conclusion

In this study, we were the first to explore the automatic identification of high-signal regions of DWI images from the description part of a head MRI report. Considering the construction of the report, we transformed the identification of high-signal regions of DWI images to an FET task and proposed an MRC-FET model. Compared with the traditional two-step FET method, the model we proposed not only simplifies the task but also has better performance, with an F1 measure that is 9% and 3. 2% higher than the F1 measures of the LSTM-FET and BERT-FET models, respectively. In the future, we will expand the dataset and use regular expressions and a more advanced pretrained language model to improve the performance of identifying the accuracy of high-signal regions of DWI images from Chinese head MRI reports.

## Data Availability

Although we obtained permission from the institutional ethics committee to use the data, we did not obtain informed consent from patients to disclose medical history data. Therefore, the data are not available. However, if you are interested in our research, you can contact xiaowei xu (xu.xiaowei@imicams.ac.cn) for further information.

## List of abbreviations

T1WI: T1-weighted imaging.
T2WI: T2-weighted imaging.
FLAIR: fluid attenuated inversion recovery.
DWI: diffusion-weighted imaging.
ADC: apparent diffusion coefficient.
EMR: electronic medical record.
NER: named-entity recognition.
FET: fine-grained entity typing.
MRC-FET: fine-grained entity typing model based on machine reading comprehension.
LSTM-FET: fine-grained entity typing model based on LSTM.
BERT-FET: fine-grained entity typing model based on BERT.
